# Concurrent Pseudogout and Parvimonas micra Prosthetic Joint Infection: Polymerase Chain Reaction (PCR) Can Be the Key to Success

**DOI:** 10.7759/cureus.86218

**Published:** 2025-06-17

**Authors:** Markus Saner, Georg Julian Claas, Randa Elsheikh, Michael Hirschman, Natalie Mengis

**Affiliations:** 1 Department of Orthopedic Surgery and Traumatology, Kantonsspital Baselland, Bruderholz, CHE; 2 Department of Clinical Research, Research Group Michael T. Hirschmann, Regenerative Medicine and Biomechanics, University of Basel, Basel, CHE; 3 Department of Medicine and Infectious Diseases Service, Kantonsspital Baselland, Bruderholz, CHE; 4 Department of Orthopedics and Traumatology, Kantonsspital Baselland, Bruderholz, CHE; 5 Department of Clinical Research, University of Basel, Basel, CHE

**Keywords:** calcium pyrophosphate deposition disease, parvimonas micra, pcr, periprosthetic joint infection, pseudogout

## Abstract

*Parvimonas micra* is an opportunistic oral pathogen; it is a fastidious gram-positive anaerobic organism that has been rarely associated with periprosthetic joint infections (PJIs). Owing to its slow growth and tendency to form biofilms, the organism often eludes conventional diagnostic approaches. We present a case of culture-negative PJI following multiple revision total knee arthroplasties, which was masked by calcium pyrophosphate deposition (CPPD) disease. Despite multiple negative cultures and empirical treatment, the patient experienced persistent symptoms and prosthetic loosening. Definitive diagnosis was only achieved through polymerase chain reaction (PCR) of periprosthetic tissue, which identified *P. micra* as the causative organism. Following the complete exchange of foreign material and targeted antimicrobial therapy, the patient experienced a full recovery, with symptom improvement and restoration of joint function. This case highlights the importance of molecular diagnostics in suspected PJI when cultures are repeatedly negative, particularly in the context of coexisting inflammatory conditions, such as CPPD. PCR and, when necessary, next-generation sequencing should be integral tools in the diagnostic algorithm for complex culture-negative PJIs.

## Introduction

The incidence of total knee arthroplasty (TKA) has increased markedly in recent years, as shown in national database studies [1], with the trend expected to continue in the next decades due to demographic shifts and the escalating incidence of osteoarthritis [2]. Despite advances in infection prevention and control practices, periprosthetic joint infections (PJIs) remain a major challenge associated with knee arthroplasty [3]. It is estimated that PJIs with septic loosening account for over 21% of revision surgeries [4]. Importantly, complications requiring revision surgery not only represent a risk for the patient but are also associated with significant healthcare-associated costs [5]. A recent analysis of the health-economic burden of PJIs in Europe revealed that periprosthetic infections resulted in a total reimbursement cost of approximately €350 million in 2019 alone [6].

PJI can be caused by various organisms, including bacteria and fungi; however, the most common is *Staphylococcus aureus*, accounting for the majority of PJIs in total hip arthroplasties and TKA, whereas rare pathogens appear to be responsible for less than 10% of the cases [7]. Atypical courses with subacute or chronic symptoms can be caused by anaerobic bacteria such as *Parvimonas micra*. The prevalence is unclear due to the small number of cases and difficulty in culturing, but is estimated to be 2%-5% [7,8].

*P. micra* is a gram-positive, anaerobic bacterium that is part of the normal flora of the human mouth and gastrointestinal tract [9]. While the organism has been occasionally identified in cases of chronic infections, including osteomyelitis and endocarditis, periodontitis, and spondylodiscitis, its role in prosthesis-associated infections is less commonly recognized [7-18]. The difficulties of growing *P. micra* in conventional microbiological cultures present a challenge for both microbiologists and clinicians [9]. Therefore, molecular biological techniques like polymerase chain reaction (PCR) are sometimes essential for its identification [12].

Calcium pyrophosphate deposition (CPPD) disease is a crystal-induced arthropathy caused by the deposition of calcium pyrophosphate dihydrate crystals in the joint cartilage and soft tissues. It most commonly affects the knee, presenting as acute pseudogout, chronic inflammatory arthritis, or resembling osteoarthritis. Clinically, CPPD is characterized by the sudden onset of pain, swelling, and erythema. In patients with joint replacements, the clinical overlap between CPPD and PJI can complicate the diagnosis, as both conditions may present with similar symptoms and laboratory findings. The presence of CPP crystals in synovial fluid can further obscure the distinction, potentially delaying treatment.

We hereby present a case of long-standing, culture-negative PJI concomitant with CPPD, where PCR played a crucial role in establishing the diagnosis and isolating the organism.

## Case presentation

A 67-year-old female patient was referred to our outpatient clinic for ongoing right-sided knee pain with the suspicion of persistent PJI after undergoing TKA nine years ago, followed by several revision surgeries.

The patient had a history of seronegative rheumatoid arthritis, for which she took nonsteroidal antirheumatic drugs. Multiple treatment attempts with conventional synthetic disease-modifying antirheumatic drugs, biological disease-modifying antirheumatic drugs, and targeted synthetic disease-modifying antirheumatic drugs were discontinued due to adverse effects or limited treatment effect. A new treatment attempt with abatacept (modulating T-cell costimulation) was planned.

**Figure 1:**
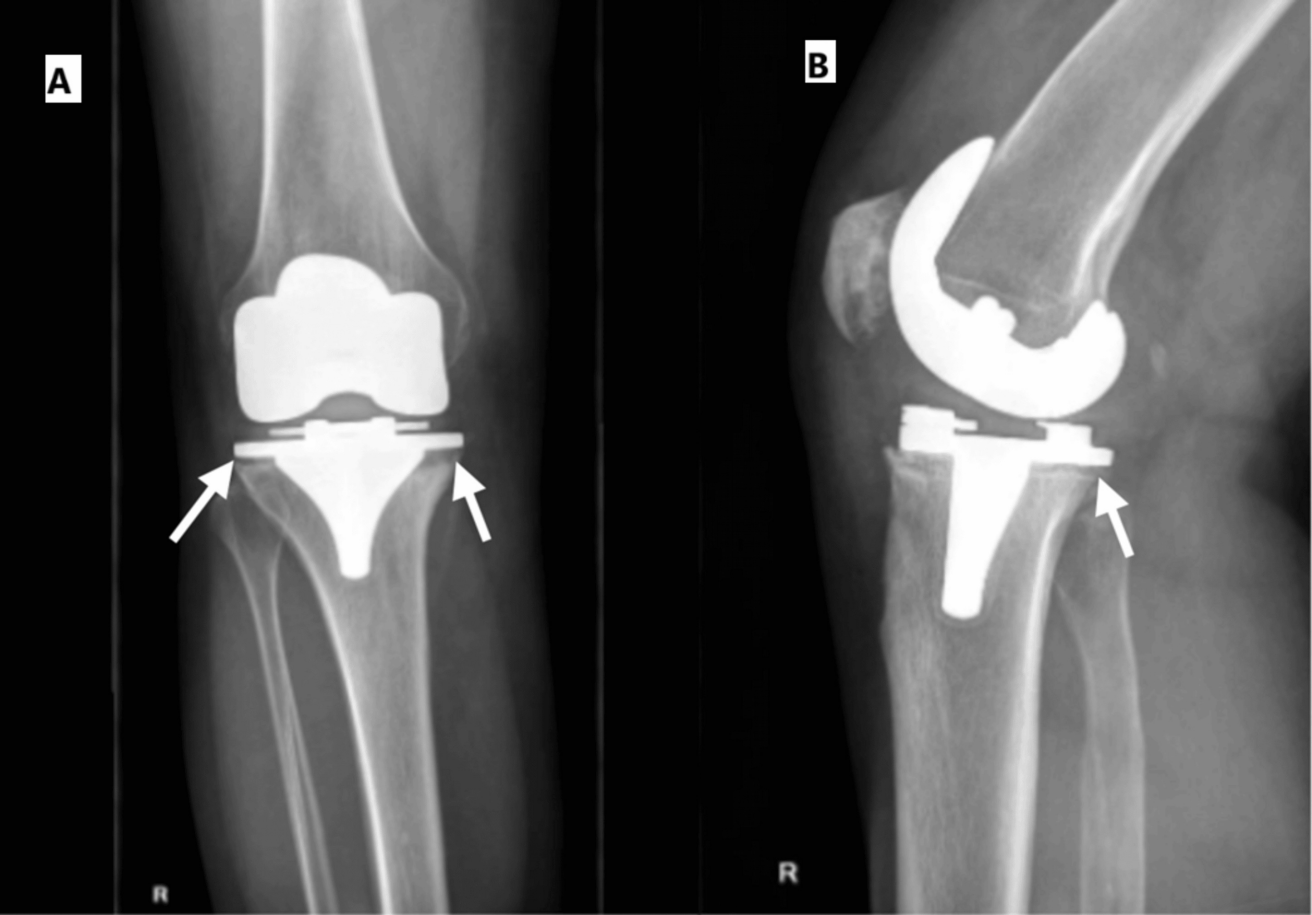
(A) Anteroposterior and (B) lateral radiographs of the right knee demonstrating loosening of the tibial component following total knee arthroplasty

The patient underwent a right TKA nine years ago for osteoarthritis. Three years later, an inlay exchange and revision of the tibial component were performed due to the aseptic loosening of the tibial component (Figure 1). The tibial component was sent for sonication, and samples were taken for histological and bacteriological analysis. Both the samples and the sonication showed no microbiological growth.

In the same year, the patient presented with persistent pain in relation to the tibial component. Knee aspiration yielded negative results, and sensitization to implant metals or bone cement components was excluded. Four years after primary TKA, the second revision with a one-stage prosthesis replacement and biopsy was performed. The biopsies again showed no evidence of bacterial growth; however, the Gram stain revealed the presence of gram-positive cocci. Nonetheless, no further diagnostics or antibiotic therapy was carried out.

Another four years later, there was an atraumatic increase in symptoms with pain, swelling, and warmth. This prompted the performance of a new aspiration, which showed a total white blood cell count of 23.7 x 10^6^/L, marked neutrophilia (97%), and many calcium pyrophosphate crystals, raising the suspicion of a PJI. Consequently, extra- and intra-articular arthrolysis, debridement, biopsy, and replacement of the retropatellar polyethylene component (due to loosening) were performed. Three weeks later, *P. micra* was detected in two of the five samples and in the sonication of the retropatellar polyethylene. Since not all components were revised and a reoperation was not feasible at the time, antibiotic therapy with 1 g of oral amoxicillin administered three times daily was initiated as suppressive therapy. After two months, the dose was halved in accordance with infectious disease recommendations, and the patient discontinued treatment independently and without replacement approximately eight months later. As a result, the patient complained of persistent knee pain during exercise and at rest, as well as fluctuating swelling and overheating.

Clinical findings

Inspection of the patient revealed a limping gait with a right-sided predominance. Standing on one leg, tiptoeing, and heel walking were not possible due to pain. The inspection of the right knee revealed an intact integument, with nonirritating wounds. Except for mild hyposensitivity in the distal lower leg, there was normal motor function. The patient could perform a straight leg raise without difficulty. However, patellar mobility was limited with positive intra-articular effusion. Furthermore, there was medial and lateral joint line tenderness, as well as pain to palpation of the pes anserinus and the iliotibial band. The range of motion in extension/flexion was 0°/5°/120°.

Diagnosis

After initial consultation at our institution, a single-photon emission computed tomography (SPECT)/computed tomography (CT) scan with technetium hydroxymethylene bisphosphonate tracer and a repeat knee aspiration were performed. The aspiration remained negative for total cell count and cell composition, as well as for Gram staining. However, the SPECT/CT showed marked bone tracer uptake around the tibial stem and rim formation at the bone-cement interface.

**Figure 2:**
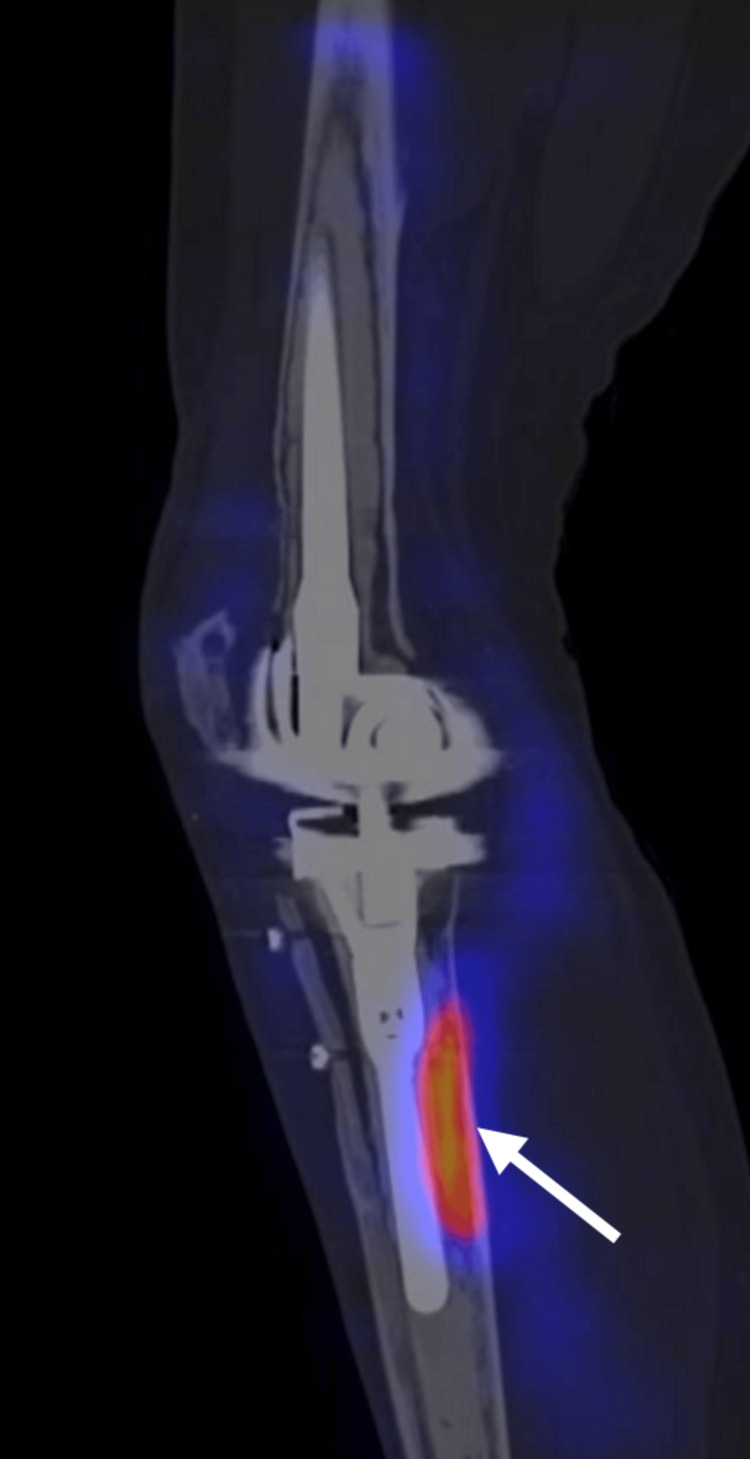
Antigranulocyte scintigraphy with SPECT of the right knee showing increased tracer uptake dorsolateral to the tibial stem 24 hours after injection SPECT: single-photon emission computed tomography

Anti-granulocyte scintigraphy with SPECT showed a clear metabolic uptake of the tracer dorsolateral to the right tibial stem 6 and 24 hours after injection (Figure 2). A decision was made to perform a knee arthroscopy with additional joint aspiration and five synovial biopsies, all of which were histomorphologically negative for acute infection. However, calcium pyrophosphate crystals were again detected.

Treatment

Given the high clinical suspicion that a persistent infection was responsible for the symptoms and findings in the SPECT/CT and antigranulocyte CT, the decision was made for a short interval two-stage revision with a vancomycin-loaded custom-made cement spacer, coupled with postoperative intravenous antibiotic therapy with 2.2 g of amoxicillin/clavulanate administered every eight hours. All of the 15 samples collected intraoperatively were negative for bacterial growth. Only one sample from the femoral shaft showed acute granulocytic inflammation in histological examination, consistent with infection. Only one of three eubacterial broad-spectrum 16S ribosomal RNA (rRNA) PCRs was able to detect Parvimonas genetic material around the tibial stem.

Importantly, although resistance testing is not possible with PCR, antimicrobial susceptibility testing performed during the arthroscopic revision procedure mentioned above showed an antibiotic-susceptible organism. Once the wound was dry, antibiotic therapy was switched to 1 g of oral amoxicillin administered every eight hours for three months after prosthesis reimplantation. Regular follow-up visits (six weeks, three months, six months, and one year) were scheduled following the final operation to ensure no signs of reinfection were present.

## Discussion

This case highlights the complexity of diagnosing and managing culture-negative periprosthetic infections, particularly when caused by rare pathogens such as *P. micra* concomitant with CPPD disease. We present a case of *P. micra* PJI following TKA, which remained undetected through conventional culture techniques across multiple revision procedures. In this case, the use of molecular biological techniques, such as PCR and eubacterial PCR, proved essential for detecting the genetic material of *P. micra* in the samples. These advanced diagnostic methods offer much higher sensitivity and specificity for detecting pathogens that are difficult to culture than traditional diagnostic techniques and are particularly valuable in cases of chronic infections with low-grade bacterial involvement [19].

*P. micra*, previously known as *Peptostreptococcus micros*, is an anaerobic, gram-positive organism that normally inhabits the oral and gastrointestinal microbiota. It is a well-known cause of periodontitis and can be implicated in the pathogenesis of spondylodiscitis, iliopsoas abscess, meningitis, endocarditis, vertebral osteomyelitis, and, less frequently, prosthetic joint infections [17]. In the literature, only 10 studies reported the occurrence of *P. micra*-associated PJI, of which six occurred following TKA (Table 1) [14,15,20-23].

**Table 1:** Parvimonas micra PJI cases reported in the literature ^*^The study reports two cases of PJI with *Parvimonas micra* CPPD: calcium pyrophosphate deposition; IV: intravenous; TKA: total knee arthroplasty; THA: total hip arthroplasty; PCR: polymerase chain reaction; NR: not reported; SPECT: single-photon emission computed tomography; CT: computed tomography; PJI: periprosthetic joint infection

Study	Sex	Age (years)	Joint	Onset of symptoms	Concomitant disease	Associated CPPD	Diagnosis	Antibiotic therapy	Surgical interventions	Outcome
Stoll et al. [22]	Female	68	Knee	4 years after TKA	Rheumatoid arthritis and Sjögren’s syndrome	No	Knee exudate aspiration culture	Six weeks of IV clindamycin and IV rifampin	Periodic needle aspiration and joint irrigation	Full recovery
Bartz et al. [24]	Female	63	Hip	9 years after THA	Dental infection	No	Surgical tissue aerobic and anaerobic culture	IV clindamycin 4 × 0.5 g/day	Prosthesis removal and one-stage exchange procedure	Full recovery
Rieber et al. [20]	Female	75	Knee	1 month after TKA	NR	No	Surgical tissue aerobic and anaerobic culture	NR	NR	NR
Bangert et al. [21]	Female	81	Knee	3 years after TKA	Periodontal disease, congestive heart failure, coronary artery disease, hypertension, type 2 diabetes, obstructive sleep apnea	Yes	Repeated synovial fluid culture	IV moxifloxacin	Prosthesis removal, one-stage exchange, and surgical debridement	Concurrent pseudogout diagnosis. Death in the immediate postoperative period due to a cardiac event
Huang et al. [25]	Female	65	Hip	7 years after THA	No	No	Metagenomic next-generation sequencing on synovial fluid	Two weeks of IV piperacillin/tazobactam followed by eight weeks of oral amoxicillin/clavulanate	Two-stage revision	Full recovery
Rieber et al. [26]	Female	78	Hip	6 months after THA	NR	NR	Surgical tissue aerobic and anaerobic culture	NR	NR	NR
Randall et al. [15]	Female	67	Knee	8 weeks after TKA	Gingivitis	No	Synovial fluid anaerobic culture	Six weeks of IV ceftriaxone	Two-stage revision	Instability requiring revision with total stabilizer implant insertion
Cao et al. [23]	Female	39	Knee	3 years after TKA	Rheumatoid arthritis	No	Knee exudate aspiration aerobic and anaerobic culture	NR	Surgical debridement	Full recovery
Anagnostakos et al. [7]	NR	NR	Hip	NR	NR	NR	Microbiological cultures and PCR	IV moxifloxacin	Two-stage revision	Full recovery
Anagnostakoset al.[7]^*^	NR	NR	Hip	NR	NR	NR	Microbiological cultures and PCR	IV ciprofloxacin	Two-stage revision	Full recovery
Piñeiro et al. [14]	Female	87	Knee	20 years after TKA	Chronic lymphocytic leukemia, diverticulosis	No	Sonication, surgical tissue aerobic and anaerobic cultures, SPECT/CT	Two weeks of IV amoxicillin-clavulanate 3 × 1 g/220 mg, eight weeks of oral amoxicillin-clavulanate 3 × 875/125 mg	Two-stage revision	Full recovery

In our case, owing to the detection of the genetic material of *P. micra*, a chronic low-grade infection was hypothesized as the cause of the patient’s chronic symptoms and prosthesis loosening. Nonetheless, no clear primary source of infection could be identified. However, hematogenous seeding from the oral cavity was the most likely route, given the patient’s history of secukinumab (Cosentyx) use, which has been associated with an increased risk of oral candidiasis and pharyngitis.

One of the major diagnostic challenges in this case was the repeated failure to isolate an organism using standard culture techniques. Prosthesis-associated infections, particularly those caused by slow-growing or fastidious organisms, are often characterized by the formation of biofilms on the implant surface. Biofilms significantly reduce the sensitivity of conventional cultures and limit the efficacy of treatment. They provide a protective niche for bacteria, shielding them from the host immune response and antibiotic therapy, therefore contributing to the chronicity of the infection [26]. In this patient, the presence of biofilm on partially retained components may have contributed to initial treatment failure and the recurrent loosening of the prosthesis.

When conventional cultures fail to identify a pathogen, molecular diagnostic tools such as 16s rRNA PCR can be invaluable. In our patient, PCR analysis of periprosthetic tissue was pivotal in detecting *P. micra* and establishing the diagnosis of PJI [27]. A similar case was reported by Dietvorst et al. [12], where a culture-negative native joint infection with *P. micra *initially revealed only calcium pyrophosphate crystals on synovial fluid analysis. Despite antibiotic therapy, the patient’s symptoms persisted, and the definitive diagnosis was ultimately established through PCR, mimicking the diagnostic pathway observed in our case [12]. Importantly, in cases with high clinical suspicion in which PCR does not yield a diagnosis, metagenomic next-generation sequencing can be particularly effective in identifying and quantifying fastidious and rare organisms [25].

The diagnostic process in this case was further confounded by the presence of calcium pyrophosphate dihydrate arthritis (CPPD), a condition known to mimic PJI. CPPD can present with acute joint pain, swelling, and elevated inflammatory markers. The identification of crystals in the synovial fluid or histological detection in the periprosthetic tissue can lead to a premature decision of an aseptic etiology. Notably, the presence of CPP crystals is the only criterion that needs to be met for diagnosing CPPD. Only one case of concurrent CPPD and PJI has been reported in the literature (Table 1). As the authors highlighted, in cases where both CPPD and PJI are suspected, the management of PJI must be prioritized, owing to the high mortality rate associated with the condition [21]. In our case, it remains unclear whether seronegative rheumatoid arthritis, not responding to any medications except nonsteroidal anti-inflammatory drugs, could be explained by CPPD.

Once *P. micra *was identified via PCR, antimicrobial therapy was initiated based on reported susceptibility profiles. Surgical management was tailored according to the patient’s clinical status and the extent of prosthetic involvement, with a two-stage exchange procedure being performed following the suspicion of biofilm persistence on retained components. The patient responded favorably to treatment, with resolution of symptoms and no signs of recurrent infection on follow-up.

This case has several important clinical implications. First, it reinforces the diagnostic value of molecular tools like PCR in detecting rare organisms like *P. micra*, where standard culturing techniques may fail to establish a diagnosis. Second, it highlights the importance of complete removal of any prosthetic device once chronic PJI is suspected to ensure complete eradication of infection and restoration of joint function. Third, it draws attention to CPPD as a potential confounder in PJI diagnosis and the importance of maintaining a high index of suspicion in atypical cases. Finally, it emphasizes the importance of a close collaboration between surgeons, infectious disease specialists, and rheumatologists in such complex cases, supporting the adoption of a multidisciplinary approach.

## Conclusions

This case underscores the critical role of PCR in diagnosing culture-negative PJI caused by rare and fastidious organisms such as *P. micra*, especially in the challenging context of concomitant CPPD. Traditional cultures repeatedly failed to identify a pathogen despite ongoing symptoms and multiple revisions. *P. micra* was ultimately detected through PCR, highlighting the value of molecular diagnostics in uncovering elusive infections that conventional methods may miss.
